# Arrhythmias in Cardiac Sarcoidosis: Pathophysiology, Diagnostic Strategies, Risk Stratification for Sudden Cardiac Death, and Contemporary Management—A Narrative Review

**DOI:** 10.3390/jcm15176619

**Published:** 2026-08-27

**Authors:** Mehdi Guedira, Jaouad Nguadi, Damien Poindron, Nicolas Lellouche, Cyrus Moini

**Affiliations:** 1 Cardiology Department, Groupe Hospitalier Sud Île-de-France (GHSIF), 270 Avenue Marc Jacquet, 77011 Melun, Francecyrus.moini@ghsif.fr (C.M.); 2 Cardiology Department, Rythmos, Clinique Les Fontaines Melun, 77000 Melun, France; 3 Cardiology Department, Rythmos, Hôpital Privé d’Antony, 92160 Antony, France; 4 Cardiology Department, Hôpital Henri Mondor, 94000 Créteil, France

**Keywords:** cardiac sarcoidosis, arrhythmias, conduction disorders, sudden cardiac death, risk stratification, implantable cardioverter-defibrillator

## Abstract

**Background**: Cardiac sarcoidosis (CS) is a potentially life-threatening manifestation of systemic granulomatous disease, characterized by a heterogeneous spectrum of arrhythmic and conduction disorders that represent the leading cause of CS-related sudden cardiac death (SCD). Clinical cardiac involvement is estimated at 5% of sarcoidosis patients, while silent myocardial infiltration is detected in 20–25% of autopsy series. Diagnosis is difficult because the disease frequently runs a subclinical course and no single test is pathognomonic. **Methods**: PubMed and Embase were searched to 5 August 2026 for this narrative review, incorporating peer-reviewed articles, international guidelines (HRS 2014, JCS 2016, AHA/ACC/HRS 2017, ESC 2022, AHA 2024, the 2024 European clinical consensus statement and the 2025 ESC guidelines on myocarditis and pericarditis), and registry data. **Results**: Atrioventricular (AV) block occurs in 23–30% of CS patients and frequently requires permanent pacing. Fatal ventricular arrhythmia rates reach 20.7% at 5 years and 31.9% at 10 years (ILLUMINATE-CS registry, *n* = 512). Late gadolinium enhancement (LGE) on cardiac magnetic resonance (CMR) performs well diagnostically (pooled sensitivity 95%, specificity 85%) and is associated with an odds ratio of 10.74 for arrhythmic events. 18F-fluorodeoxyglucose positron emission tomography (FDG-PET) provides complementary value for guiding immunosuppressive therapy and pre-ablation risk assessment. Implantable loop recorders enable early arrhythmia detection in patients not yet meeting implantable cardioverter-defibrillator (ICD) criteria. **Conclusion**: Optimal management of arrhythmias in CS requires a multimodal diagnostic approach integrating electrocardiography, advanced cardiac imaging, and continuous rhythm monitoring. Risk stratification for SCD remains the central challenge, requiring individualized decision-making that integrates left ventricular ejection fraction (LVEF), imaging biomarkers, and electrophysiological data. Future prospective studies should aim to refine predictive risk algorithms, assess the role of emerging immunomodulatory agents such as JAK/STAT inhibitors, and validate artificial intelligence (AI)-assisted tools intended to identify cardiac involvement and arrhythmic risk earlier in the disease course.

## 1. Introduction

Sarcoidosis is a multisystem inflammatory disorder defined histologically by non-caseating granulomas, with the lungs and lymph nodes most often involved. Cardiac disease is clinically apparent in roughly one patient in twenty, yet autopsy series place the true frequency of myocardial infiltration between 20% and 25%—a discrepancy that underlies much of the diagnostic difficulty in this field [1].

The cardiac phenotype of cardiac sarcoidosis (CS) is broad, encompassing conduction disease, ventricular arrhythmia, heart failure and, less commonly, valvular or pericardial involvement. Arrhythmic presentations dominate both in frequency and in severity, typically as atrioventricular block, ventricular tachycardia, or sudden cardiac death (SCD). Cardiac involvement ranks second only to pulmonary disease among causes of death in sarcoidosis [2], which is why early recognition and individualised management matter.

Symptoms range from palpitations to syncope, but a substantial proportion of patients remain asymptomatic until a serious event supervenes. Managing rhythm disorders in this setting is inherently multidisciplinary: immunosuppression to control granulomatous inflammation, antiarrhythmic drugs, implantable devices, and catheter ablation in selected patients.

Early identification and appropriate management of these rhythm disturbances are therefore essential to improve patient prognosis.

This narrative review aims to provide a comprehensive, clinically oriented overview of the arrhythmic manifestations of cardiac sarcoidosis and to highlight the central role of the cardiac electrophysiologist in the diagnostic and therapeutic management of this multifaceted condition.

**Search strategy and evidence selection.** We searched PubMed and Embase from database inception to 5 August 2026, without language restriction. The search combined the terms “cardiac sarcoidosis” or “myocardial sarcoidosis” with “arrhythmia”, “ventricular tachycardia”, “atrioventricular block”, “sudden cardiac death”, “implantable cardioverter-defibrillator”, “catheter ablation”, “cardiac magnetic resonance”, “positron emission tomography” and “risk stratification”. Reference lists of retrieved articles, international guidelines and consensus statements were screened manually for additional sources.

Records were screened by title and abstract, and potentially relevant articles assessed in full text. We included original research, systematic reviews, meta-analyses, international guidelines and consensus statements, and registry data. Case reports were included only where they illustrated a diagnostic or therapeutic scenario not otherwise documented.

Evidence was prioritised hierarchically: contemporary international guidelines and consensus statements first, followed by multicentre registries and large cohorts, then single-centre series. Given the absence of randomised controlled trials in cardiac sarcoidosis, observational data and expert consensus necessarily underpin most current recommendations, and are cited as such throughout. Where recommendations rest on expert opinion rather than empirical data, this is stated explicitly.

Where several publications reported overlapping cohorts, the most complete or most recent publication was retained; conference abstracts were replaced by the corresponding full publication wherever one existed. Where guideline recommendations differed between frameworks, the primary documents were consulted directly rather than relying on secondary citation.

This is a narrative review. It was not registered, and no formal risk-of-bias assessment or quantitative synthesis was undertaken.

## 2. Pathophysiological Mechanisms of Arrhythmogenesis in Cardiac Sarcoidosis

Arrhythmogenesis in CS results from two distinct groups of mechanisms: those arising directly from granulomatous myocardial involvement, and those mediated indirectly by the haemodynamic consequences of ventricular dysfunction. Distinguishing between them is clinically relevant, since the former may be reversible under immunosuppression whereas the latter generally are not.

### 2.1. Direct Mechanisms of Granulomatous Involvement

**The inflammatory phase.** Granuloma formation reflects a dysregulated T-cell response with upregulation of interferon-γ, tumour necrosis factor-α (TNF-α), transforming growth factor-β (TGF-β), interleukin-2 (IL-2) and interleukin-12 (IL-12), culminating in macrophage activation and the development of non-necrotizing (non-caseating) granulomas [3,4,5]. These lesions show a predilection for the basal interventricular septum, where the atrioventricular (AV) node and His bundle reside, accounting for the frequency of conduction disturbances at presentation.

During this phase, arrhythmogenesis is driven predominantly by triggered activity and abnormal automaticity. Inflammatory cytokines modulate ion channel function and calcium handling, lowering the threshold for early and delayed afterdepolarizations. This mechanistic profile explains why ventricular ectopy and non-sustained ventricular tachycardia occurring during active inflammation may respond to immunosuppressive therapy.

**The fibrotic phase.** As inflammation subsides, granulomas are replaced by patchy fibrosis interspersed with surviving myocyte bundles. This architecture generates zones of slow conduction and unidirectional block—the substrate for macro-reentrant ventricular tachycardia. Unlike the inflammatory phase, this substrate is fixed and does not regress with immunosuppression, which explains why established scar-related ventricular tachycardia often requires device therapy or catheter ablation rather than anti-inflammatory treatment alone [4].

**Autonomic dysfunction.** Granulomatous infiltration and subsequent fibrosis may damage intramyocardial sympathetic and parasympathetic nerve endings. Regional denervation could theoretically produce heterogeneous adrenergic sensitivity across the myocardium, thereby increasing dispersion of repolarization and facilitating catecholamine-sensitive arrhythmias. However, data specific to CS remain scarce, and the clinical relevance of autonomic remodelling in this setting has yet to be established.

### 2.2. Indirect, Haemodynamically Mediated Mechanisms

Beyond direct tissue involvement, arrhythmias in CS may arise from the consequences of ventricular dysfunction itself. Progressive systolic impairment and elevated filling pressures promote chamber dilatation, wall stress and secondary neurohormonal activation—mechanisms shared with other dilated cardiomyopathies and independent of granulomatous burden.

This distinction is particularly relevant for atrial arrhythmias. Atrial fibrillation (AF) in CS may follow two separate pathways. **Direct atrial involvement** produces inflammation and scarring of atrial myocardium; supporting this, atrial 18F-FDG uptake has been identified as an independent predictor of incident AF [6]. **Indirect atrial remodelling** arises from chronically elevated left atrial pressure secondary to left ventricular dysfunction, producing stretch-induced fibrosis and conduction slowing. These converging processes correspond to the concept of atrial cardiomyopathy, in which structural, contractile and electrophysiological atrial changes constitute the substrate for AF regardless of the initiating insult [7]. In practice the two pathways frequently coexist, and their relative contribution influences whether AF is likely to respond to immunosuppression.

In a cohort of 118 CS patients without prior atrial fibrillation, 34 (29%) developed paroxysmal AF over a median follow-up of three years [6].

### 2.3. Distribution of Involvement

Myocardial involvement is typically multifocal. The left ventricle is most frequently affected, particularly the basal septum and papillary muscles, but right ventricular and atrial involvement are also well described [8]. This anatomical heterogeneity underlies the wide clinical spectrum observed, ranging from isolated conduction disease to ventricular arrhythmias and heart failure.

## 3. Clinical Red Flags: When to Suspect Cardiac Sarcoidosis

Several presentations should raise the possibility of CS: high-degree atrioventricular block of unexplained cause in a patient under sixty; ventricular arrhythmia without identifiable substrate; unexplained impairment of left ventricular systolic function; or structural findings such as a thinned basal septum or a focal aneurysm in the absence of coronary disease.

### 3.1. ECG and Ambulatory Monitoring

The twelve-lead electrocardiogram is insensitive but indispensable, since it is often the investigation that first raises suspicion. Conduction delay is the commonest abnormality, with complete atrioventricular block reported in 23–30% of cases and attributable to granulomatous infiltration and fibrosis of the basal septum [9]. Fragmented QRS complexes, right or left bundle branch block reflecting intraventricular infiltration, and supraventricular arrhythmia may all be seen. Niemelä and colleagues followed 118 patients with CS and no prior atrial fibrillation: over a median of three years, 34 (29%) developed paroxysmal atrial fibrillation, seven progressed to persistent and four to permanent forms. Atrial 18F-FDG uptake emerged from that study as an independent predictor [6].

At ventricular level, the ILLUMINATE-CS registry followed 512 patients diagnosed between 2001 and 2017 and reported fatal ventricular arrhythmia in 20.7% by five years and 31.9% by ten [10]. Because Japanese criteria treat high-degree block and sustained ventricular tachycardia or fibrillation as major diagnostic features while relegating non-specific abnormalities to minor status, serial electrocardiography is warranted, supplemented by extended ambulatory monitoring—conventional Holter recording or, where the clinical course suggests progression, an implantable loop recorder [11].

Separating CS from arrhythmogenic right ventricular cardiomyopathy (ARVC, formerly termed arrhythmogenic right ventricular dysplasia) or from myocarditis can be difficult without histology. An electrocardiographic algorithm has been proposed for patients presenting with ventricular tachycardia of right bundle branch block morphology. In ARVC, diffuse fibrofatty replacement of the right ventricle produces generalised conduction slowing and globally reduced voltages; in CS, granulomas create discrete zones of block while surrounding voltages remain comparatively preserved, which manifests as an R′ wave of greater amplitude.

Combining a PR interval of at least 220 ms, the presence of an R’ wave, and a maximal R’ wave area of 1.65 mm^2^ or more across leads V1 to V3 yielded a sensitivity of 85% and a specificity of 96% for CS [12]. The electrocardiographic and ambulatory monitoring findings that should raise suspicion of CS are summarised in Table 1.

### 3.2. Echocardiography

Echocardiography is usually performed early in the diagnostic pathway, but its yield in CS is limited: sensitivity is modest and specificity low, so that a normal study cannot exclude the disease. Reported abnormalities include regional wall motion disturbance, thinning of the basal interventricular septum, ventricular dilatation, aneurysm formation, and impairment of systolic or diastolic function in either chamber. A characteristic feature is that wall motion abnormalities do not respect coronary territories. Basal septal thinning and localised aneurysms, although strongly suggestive when present, are found in only a minority of published series [4].

These findings feature among the diagnostic criteria discussed in Section 4. Diagnostic yield improves when echocardiographic abnormalities are interpreted alongside electrocardiographic or ambulatory monitoring findings rather than in isolation. Left ventricular ejection fraction remains among the most robust prognostic markers in CS and guides much of subsequent management. Global longitudinal strain has more recently emerged as a means of identifying myocardial involvement in patients whose ejection fraction is still preserved [14]. Nevertheless, a proportion of patients present with an entirely normal electrocardiogram and echocardiogram, and in these cases cardiac magnetic resonance (CMR) and 18F-fluorodeoxyglucose positron emission tomography (FDG-PET) become the decisive investigations.

### 3.3. Cardiac Magnetic Resonance

CMR is a cornerstone of CS diagnosis, with a pooled sensitivity of 95% and specificity of 85% in meta-analysis [15]. These figures should be interpreted with caution, as diagnostic accuracy varies according to the population studied, the imaging protocol, patient preparation and, critically, the reference standard against which the technique is compared—a limitation shared by all imaging modalities in a disease lacking a true gold standard.

**What late gadolinium enhancement does and does not indicate.** Gadolinium is an extracellular contrast agent, rapidly cleared from healthy myocardium but retained where the extracellular space is expanded. Late gadolinium enhancement (LGE) therefore reflects myocardial injury and expansion of the extracellular compartment, whether due to fibrosis, inflammatory infiltration, or both. It is not a specific marker of active inflammation, and its presence alone cannot distinguish an actively inflamed segment from established scar [16].

This distinction has direct therapeutic consequences: inflammation may respond to immunosuppression whereas fibrosis will not. Discriminating between them therefore requires complementary techniques.

**Distinguishing inflammation from fibrosis.** T2-weighted imaging and T2 mapping detect myocardial oedema and are the CMR techniques best suited to identifying active inflammation. Native T1 mapping and extracellular volume quantification are sensitive to both oedema and fibrosis and must be interpreted alongside T2-based sequences. FDG-PET provides an independent metabolic readout of inflammatory activity. In practical terms, a segment showing LGE with elevated T2 signal or FDG uptake is likely to harbour active inflammation, whereas LGE with normal T2 signal and no FDG uptake indicates fibrosis without ongoing inflammatory activity [17].

**Figure 1:**
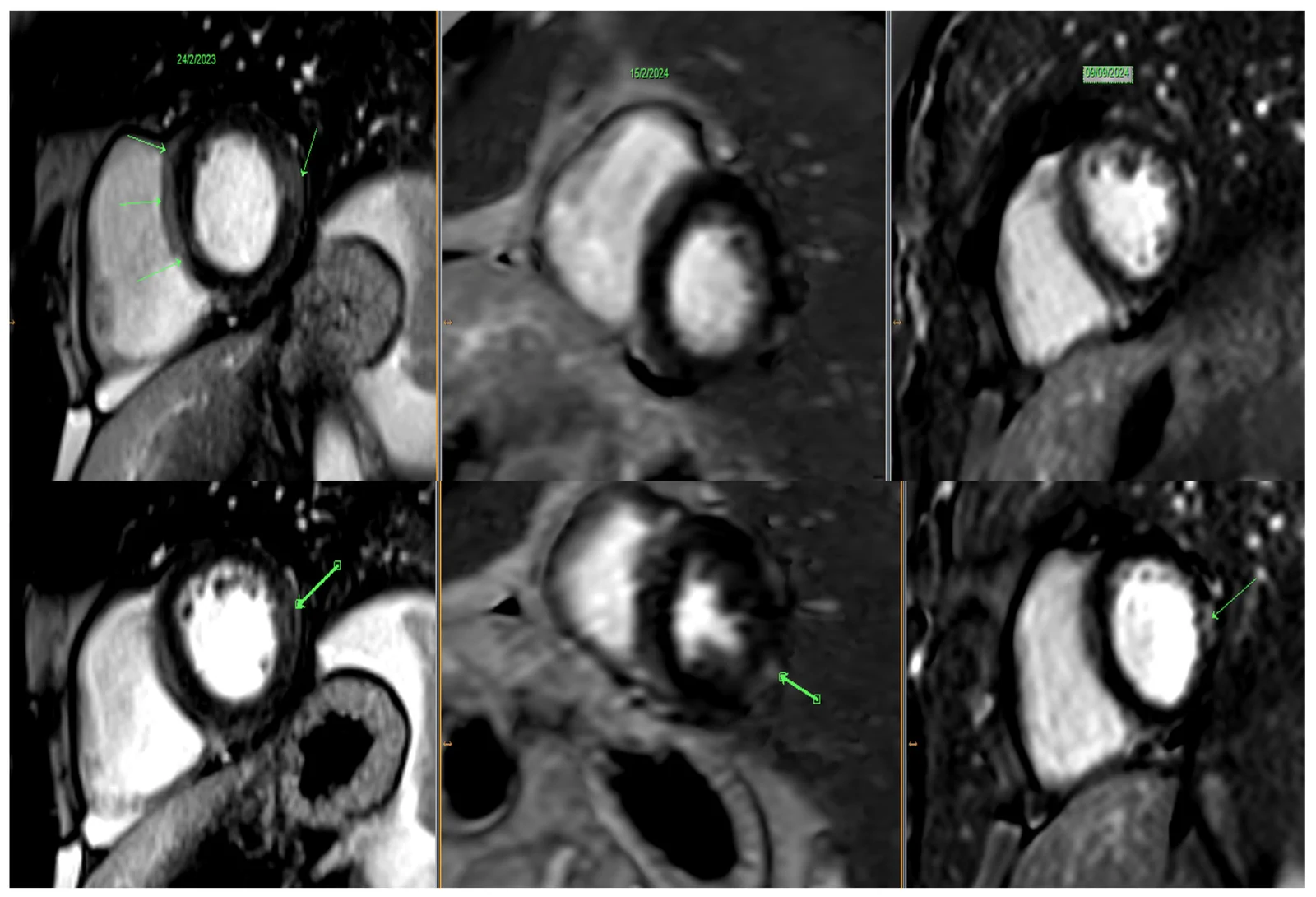
Cardiac magnetic resonance (phase-sensitive inversion recovery [PSIR] late gadolinium enhancement sequence): extensive late gadolinium enhancement of the interventricular septum at the right ventricular junction and anterior wall, with an additional subepicardial lesion in the mid-lateral wall. Sequential imaging at 12 and 18 months after initiation of immunosuppressive therapy shows a reduction in the extent of septal enhancement. Since LGE reflects expansion of the extracellular space rather than inflammatory activity specifically, and since serial LGE quantification is sensitive to differences in acquisition and analysis technique, this change should be interpreted with caution and considered alongside clinical status and complementary markers of inflammatory activity. Green arrows indicate the areas of late gadolinium enhancement discussed in the text. Images are displayed in greyscale; the acquisition date of each study appears in the upper part of each panel.

**Complementary roles of CMR and FDG-PET.** The two modalities interrogate different aspects of the disease and are complementary rather than interchangeable. CMR excels at characterising tissue structure, detecting fibrosis and quantifying its extent, and offers superior spatial resolution; FDG-PET is superior for detecting and monitoring active inflammation. Discordant findings are not uncommon and carry clinical meaning: an LGE-positive, PET-negative pattern suggests burnt-out disease, whereas PET positivity without LGE may indicate early inflammatory involvement preceding structural damage. Combined assessment improves diagnostic confidence and is recommended in current consensus documents [5,17]. An illustrative example of septal late gadolinium enhancement and its evolution under immunosuppressive therapy is shown in Figure 1.

### 3.4. FDG Positron Emission Tomography (FDG-PET)

FDG-PET identifies metabolically active inflammatory tissue. Activated macrophages and lymphocytes take up 18F-fluorodeoxyglucose through the GLUT1 and GLUT3 transporters, whereas cardiomyocytes rely on the insulin-dependent GLUT4 transporter. This difference can be exploited: prolonged fasting or a high-fat, carbohydrate-restricted preparation shifts myocyte metabolism towards fatty acid oxidation and suppresses physiological myocardial uptake, so that inflammatory foci become conspicuous [18,19]. Adequate dietary preparation is therefore critical, and inadequate suppression is a common cause of uninterpretable studies. When focal uptake is combined with a corresponding perfusion defect and evidence of extracardiac disease, reported sensitivity approaches 83% with specificity near 100% [20].

### 3.5. Endomyocardial Biopsy and Guided Strategies

Because granulomatous involvement is patchy and unevenly distributed, unguided endomyocardial biopsy samples the affected tissue in only about one case in five. Directing the bioptome towards abnormalities identified on FDG-PET or CMR raises the yield to roughly half. In practice, histological confirmation is frequently obtained from an extracardiac site instead, most often a lymph node or the lung [5]. Contemporary criteria, notably those issued by the Japanese societies, accommodate this reality by allowing imaging to substitute for myocardial histology in defined circumstances, including isolated cardiac disease.

Two recent studies refine this picture. In a Finnish series, the diagnostic performance of endomyocardial biopsy in CS was examined systematically, confirming that yield remains the principal constraint on the histological pathway [21]. From the Japanese ILLUMINATE-CS registry, an analysis of predictors and outcomes associated with a positive biopsy found that histological confirmation carries prognostic as well as diagnostic significance, and identified clinical features associated with a positive result [22]. Together these findings support a selective rather than routine approach: biopsy is most informative when imaging has already localised a target and when histological confirmation would alter management.

### 3.6. Three-Dimensional Electroanatomical Mapping

Coupling endomyocardial biopsy to three-dimensional electroanatomical mapping offers a way of improving histological yield. The limited return of fluoroscopy-guided right ventricular sampling reflects both the scattered distribution of granulomas and the tendency of CS to involve the mid-myocardial and subepicardial layers of the left ventricle preferentially.

The largest reported experience of electrogram-guided sampling comes from Ezzeddine and colleagues, who used a three-dimensional mapping system to biopsy the right and/or left ventricle, frequently in the same session as ablation of ventricular tachycardia or premature ventricular complexes. Target sites were selected on the basis of abnormal bipolar signals—fractionated electrograms, late potentials or reduced voltage. Fluoroscopy and intracardiac echocardiography were used to direct the bioptome towards these regions. Where no abnormal endocardial electrogram was found, sampling was guided instead by CMR or PET findings. Abnormal electrogram signals predicted CS with a sensitivity of 89% but a specificity of only 33% [23].

### 3.7. Continuous Rhythm Surveillance After Diagnosis

The modalities described above serve to establish or exclude a diagnosis of CS. The tools discussed in this section serve a different purpose: once the diagnosis is made, continuous rhythm surveillance detects arrhythmic events that inform risk stratification and therapeutic decisions over time. They are presented here for continuity with the preceding discussion of rhythm monitoring, but their role is one of longitudinal management rather than diagnosis.

#### 3.7.1. Implantable Loop Recorders

Implantable loop recorders (ILRs) provide continuous rhythm surveillance in CS patients who do not meet criteria for implantable cardioverter-defibrillator (ICD) implantation.

In a retrospective study of 547 patients with extracardiac sarcoidosis systematically screened for cardiac involvement, 105 patients with CS were stratified by a multidisciplinary team and followed for a mean of 33 ± 16 months. An ICD was implanted in 17 high-risk patients (16.2%), while 80 low-risk patients (76.1%) received an ILR. The primary endpoint—sustained ventricular arrhythmia, appropriate ICD therapy, or cardiac death—occurred in 4.8% of patients, corresponding to an annualized event rate of 1.7%: 9.8% in the high-risk group versus 0.4% in the low-risk group. During follow-up, nine low-risk patients received an ICD, in seven cases prompted by ILR findings [24].

These data support two conclusions. First, multidisciplinary risk stratification effectively separates patients with markedly different event rates. Second, ILR monitoring detects clinically relevant arrhythmias in patients initially classified as low risk, prompting reclassification and device implantation in a minority.

**The prognostic value of this reclassification remains unproven.** None of the patients who received an ICD following ILR findings subsequently required device therapy during follow-up, and the authors explicitly concluded that ILR monitoring enabled early arrhythmia detection without demonstrating an effect on prognosis [24]. Current evidence therefore supports ILRs as a tool for refining risk assessment rather than as an intervention shown to improve survival.

Consensus documents suggest that ILRs may be particularly useful in patients with suspected CS presenting with unexplained syncope, palpitations, or conduction abnormalities who do not yet meet ICD criteria [17,25]. Whether earlier detection translates into improved outcomes will require prospective evaluation.

#### 3.7.2. Wearable Devices

Wearable devices, particularly smartwatches with single-lead electrocardiographic capability, are increasingly used for the detection of cardiac arrhythmias. In general populations they detect atrial fibrillation with a sensitivity of 96% and specificity of 91%, and complete bundle branch block with a sensitivity of 85% and specificity of 98%; multi-lead configurations further improve the detection of repolarisation abnormalities and the differentiation of bradyarrhythmias [26].

CS-specific data are lacking. No study has evaluated the diagnostic yield of wearable monitoring in a CS cohort, and the performance figures cited above derive from unselected populations in which the pre-test probability of conduction disease differs substantially from that of CS. Furthermore, as with implantable monitors, improved arrhythmia detection has not been shown to translate into improved outcomes.

Wearable devices should therefore be regarded as adjunctive rather than alternative to established ambulatory monitoring strategies. They may have a supplementary role in symptomatic patients between scheduled assessments, or in those declining or awaiting implantable monitoring, but they do not replace formal Holter monitoring or implantable loop recorders where these are indicated. Prospective evaluation in CS cohorts is warranted.

## 4. Diagnostic Criteria and Clinical Pathways: A Comparative Appraisal

Five major documents currently inform the diagnosis of CS: the HRS expert consensus statement (2014) [25], the JCS guidelines (2016) [27], the AHA Scientific Statement (2024) [5], the European clinical consensus statement (2024) [17], and the 2025 ESC guidelines on myocarditis and pericarditis [28]. They share a common histological standard but diverge substantially in how a diagnosis may be established without myocardial tissue—a divergence with direct consequences for which patients are identified and treated.

### 4.1. The Histological Standard

All five frameworks agree that demonstration of non-caseating granulomas on myocardial tissue, obtained by endomyocardial biopsy or surgical specimen, establishes the diagnosis when alternative causes are excluded. In practice this pathway is limited by the low yield of unguided biopsy, discussed in Section 3.5.

### 4.2. The HRS Clinical Pathway and Its Principal Limitation

The HRS pathway allows a probable diagnosis without myocardial tissue, but only in patients whose extracardiac sarcoidosis has been proven histologically, and only when at least one supporting feature is present [25]. Those features are: a cardiomyopathy or heart failure syndrome that improves on corticosteroids or other immunosuppression; systolic impairment below 40% for which no alternative explanation exists; sustained ventricular tachycardia, whether spontaneous or provoked, again unexplained; second-degree Mobitz II or complete atrioventricular block; patchy myocardial tracer uptake on PET in a pattern consistent with the disease; myocardial enhancement on CMR in a comparable pattern; or gallium avidity in a compatible distribution.

The requirement for histologically proven extracardiac disease constitutes the principal limitation of this framework. **Patients with isolated cardiac sarcoidosis—in whom no extracardiac focus exists to biopsy—cannot be diagnosed through this pathway.** They are left dependent on myocardial biopsy, with its recognised sampling limitations, and are consequently at risk of remaining undiagnosed or of being labelled as arrhythmogenic cardiomyopathy or idiopathic dilated cardiomyopathy.

### 4.3. The JCS Framework and the Recognition of Isolated Disease

The JCS criteria address this gap directly. Alongside a pathway analogous to the HRS one, they permit diagnosis on the basis of clinical findings strongly suggestive of pulmonary or ophthalmic sarcoidosis combined with at least two major criteria, or one major and two minor criteria [27].

**Major criteria:** high-grade atrioventricular block, including complete block, or fatal ventricular arrhythmia such as sustained ventricular tachycardia or ventricular fibrillation; basal thinning of the ventricular septum or other abnormal ventricular wall anatomy, including ventricular aneurysm or regional wall thickening; left ventricular contractile dysfunction with left ventricular ejection fraction (LVEF) below 50%, or focal wall asynergy; abnormally high tracer accumulation in the heart on 67Ga scintigraphy or 18F-FDG PET; delayed myocardial contrast enhancement on gadolinium-enhanced CMR.

**Minor criteria:** abnormal ECG findings, comprising ventricular arrhythmias (non-sustained ventricular tachycardia, multifocal or frequent premature ventricular complexes), bundle branch block, axis deviation, or abnormal Q waves; perfusion defects on myocardial perfusion single-photon emission computed tomography (SPECT); endomyocardial biopsy showing monocyte infiltration with moderate to severe interstitial fibrosis.

Cardiac involvement is considered strongly suggested when two or more major criteria are satisfied, or when one major criterion is accompanied by at least two of the three minor criteria [27]. The clinical diagnosis pathway additionally requires at least two of the five characteristic laboratory findings of sarcoidosis, namely bilateral hilar lymphadenopathy, elevated serum angiotensin-converting enzyme or lysozyme, elevated soluble interleukin-2 receptor, significant tracer accumulation on 67Ga scintigraphy or 18F-FDG PET, or a bronchoalveolar lavage lymphocyte CD4/CD8 ratio above 3.5 [27].

**Isolated cardiac sarcoidosis.** Most importantly for the electrophysiologist, the JCS framework provides a dedicated diagnostic pathway for disease confined to the heart—a scenario the HRS pathway cannot accommodate. Three prerequisites must first be satisfied: absence of clinical features of sarcoidosis in any organ other than the heart, following detailed assessment for respiratory, ophthalmic and cutaneous involvement; absence of extracardiac tracer uptake on 67Ga scintigraphy or 18F-FDG PET; and a chest CT showing neither lymphatic-tract opacities nor hilar or mediastinal lymphadenopathy exceeding 10 mm in short axis [27].

Once these prerequisites are met, diagnosis may be established histologically, through demonstration of non-caseating epithelioid granulomas on endomyocardial biopsy or surgical specimen. Alternatively, a clinical diagnosis is permitted when abnormal 67Ga or 18F-FDG cardiac uptake is present together with at least three of the remaining major criteria [27].

Two practical provisions of this framework deserve emphasis. First, the guidelines explicitly acknowledge diagnostic uncertainty by identifying an intermediate category of patients who should be suspected of isolated CS without yet meeting full criteria. Second —and of direct relevance to arrhythmia management—they state that when isolated CS is strongly suspected and carries a risk of death, empirical corticosteroid or immunosuppressive therapy may be initiated before diagnostic confirmation [27]. This provision is clinically important, since waiting for histological certainty in a patient with high-grade block or ventricular arrhythmia may entail unacceptable risk.

Alternative diagnoses must be excluded, in particular coronary artery disease, chronic myocarditis, giant cell myocarditis, and myocarditis associated with systemic disorders [27]. This divergence in the handling of isolated disease is the most substantive difference between the two frameworks and explains why isolated CS is recognised more frequently in Japanese series than in Western cohorts.

### 4.4. The 2024 Documents: From Binary Criteria Towards Probabilistic Assessment

Both 2024 statements move away from the notion of a single diagnostic threshold. The AHA Scientific Statement emphasises maintaining a high index of suspicion in patients presenting with new-onset heart failure, high-grade conduction disease, ventricular arrhythmias, or known extracardiac sarcoidosis, and notes that most patients with cardiac manifestations also have extracardiac disease [5]. It underlines that echocardiography has limited sensitivity and specificity, positioning advanced imaging as central rather than confirmatory.

The European clinical consensus statement similarly frames diagnosis as an integrative process combining clinical presentation with the results of complementary investigations, with explicit emphasis on multidisciplinary assessment [17]. Both documents acknowledge that the absence of randomised evidence means recommendations rest on cohort data and expert opinion, and both note substantial variation in practice between centres [5].

For the practising electrophysiologist, the operational consequence is that a diagnosis of CS is better understood as a **graded probability**—informed by clinical presentation, imaging phenotype, and response to therapy—than as a binary determination made at a single point in time.

### 4.5. Where the ESC Guidelines Apply

The 2022 ESC guidelines on ventricular arrhythmias do not provide diagnostic criteria for CS; they address management once the diagnosis is established, and are therefore considered in Section 6 [29].

### 4.6. The 2025 ESC Guidelines on Inflammatory Myopericardial Syndromes

The 2025 ESC guidelines on myocarditis and pericarditis introduce a unified framework of inflammatory myopericardial syndromes and include a dedicated set of recommendations for sarcoid myocarditis, alongside separate recommendations addressing the management of arrhythmias and the prevention of sudden cardiac death in myocarditis more broadly [28]. Their relevance here is twofold. First, they position CS explicitly within the spectrum of immune-mediated myocarditis rather than treating it as an isolated entity, which has consequences for the diagnostic pathway a patient enters when presenting with an inflammatory phenotype. Second, they address the role of advanced imaging directly, indicating that FDG-PET may be considered where CMR is inconclusive or contraindicated, particularly when CS is suspected [28]. For the electrophysiologist, this means that a patient presenting with ventricular arrhythmia and myocardial inflammation may now be evaluated through either the CS-specific frameworks discussed above or the broader myocarditis pathway, and awareness of both is necessary to avoid diagnostic delay.

## 5. Therapeutic Management: The Cardiac Electrophysiologist’s Arsenal

### 5.1. Immunosuppressants

Immunosuppression remains the foundation of treatment in CS. Its purpose is to suppress granulomatous inflammation and thereby slow disease progression and relieve symptoms. Corticosteroids are used first line; while a consistent survival benefit has not been demonstrated, symptomatic improvement is well described and the benefit appears greater when treatment begins before irreversible myocardial damage has occurred [2,30]. Response is monitored through serial imaging, using gallium-67 scintigraphy or 18F-FDG PET, alongside clinical course, and treatment is often continued for prolonged periods [27].

The indication for corticosteroid therapy in the management of arrhythmias should be guided by the presumed arrhythmic mechanism rather than applied uniformly. Arrhythmias arising during active inflammation—including high-grade atrioventricular block, ventricular ectopy and non-sustained ventricular tachycardia—may improve or resolve with immunosuppression. In contrast, established scar-mediated ventricular tachycardia arises from a fixed fibrotic substrate and is unlikely to respond to anti-inflammatory therapy alone; in these patients immunosuppression may limit further disease progression but should not be expected to suppress the arrhythmia itself.

The JCS guidelines assign a Class I recommendation to corticosteroid therapy when cardiac involvement is confirmed by positive 67Ga scintigraphy or 18F-FDG PET (evidence level 4a, grade A), and a Class IIa recommendation when cardiac involvement is not confirmed by these imaging modalities (evidence level 4b, grade C1) [27]. This gradation itself reflects the mechanistic principle above: the strength of the recommendation follows the demonstration of active inflammation.

When corticosteroids alone prove insufficient, or when adverse effects limit their use, second-line agents may be added or substituted. Cyclophosphamide, ciclosporin, azathioprine, methotrexate, thalidomide and hydroxychloroquine have all been employed, either alone or in combination with corticosteroids [30,31].

For disease refractory to both corticosteroids and second-line agents, biological therapy with infliximab, a monoclonal antibody directed against TNF-α, has been reported with encouraging results [31].

Comparative data between second-line agents have until recently been lacking. A comparative effectiveness analysis of disease-modifying antirheumatic drugs in CS now provides a basis for choosing between them rather than relying on extrapolation from extracardiac disease [32].

The duration of therapy is a further unresolved question. Treatment has conventionally been continued for prolonged periods, but a prospective cohort has reported durable remission in CS patients following discontinuation of methotrexate, indicating that indefinite immunosuppression may not be necessary in all patients [33]. Decisions to withdraw therapy should nonetheless be individualised and accompanied by imaging and rhythm surveillance, given the potential for relapse.

The effect of immunosuppression on conduction disease has been examined in a pooled analysis of thirteen observational studies encompassing 199 patients with high-grade atrioventricular block. Immunosuppressive treatment, most often corticosteroids, was given to 178 of them (89%). Atrioventricular conduction recovered in 76 of these treated patients (42%), whereas no recovery was observed in any untreated patient [34].

### 5.2. Antiarrhythmic Drugs (AAD)

The choice of antiarrhythmic drug (AAD) should be guided by cardiac function, the inflammatory activity of cardiac sarcoidosis, and the indications for device-based therapy. Notably, no antiarrhythmic agent carries a Class I recommendation in CS, reflecting the absence of randomised data in this population.

Ventricular arrhythmia in CS most often arises within scarred myocardium through reentry [35], which argues for beta-blockade and for agents that lengthen repolarization such as amiodarone and sotalol [27]; once heart failure supervenes, beta-blockers are preferred [27].

The JCS 2016 guidelines stratify recommendations according to whether ventricular tachycardia is controllable. Amiodarone, sotalol and class Ib agents are reasonable for ventricular tachycardia refractory to control (Class IIa), whereas amiodarone, sotalol and verapamil carry only a Class IIb recommendation once ventricular tachycardia is adequately controlled. Class Ia and Ic agents may be considered as a last resort in refractory ventricular tachycardia (Class IIb) but are not recommended in adequately controlled ventricular tachycardia (Class III), owing to their proarrhythmic potential in scarred myocardium [27]. These recommendations are summarised in Table 2.

Amiodarone warrants particular caution in this population. Given the high prevalence of concomitant pulmonary sarcoidosis, its potential pulmonary toxicity may be difficult to distinguish from progression of the underlying granulomatous lung disease, and close respiratory monitoring is advised [17].

The use of beta-blockers may appear paradoxical in a disease characterised by a high incidence of conduction disease. Two considerations may help reconcile this apparent contradiction. Atrioventricular block in CS is frequently infra-Hisian, reflecting granulomatous infiltration and fibrosis of the His-Purkinje system rather than nodal slowing, and beta-blockade acts principally on nodal conduction; the clinical relevance of this distinction in CS has not, however, been formally studied. In addition, a substantial proportion of patients with conduction disease will already carry a pacemaker or a defibrillator with pacing capability, which removes the bradycardic constraint on therapy. In patients without a device and with evidence of conduction disease, beta-blockers should be introduced cautiously and with electrocardiographic surveillance.

Ventricular arrhythmia can often recur, even after a successful ablation procedure, which justifies the continuation of AAD after ablation [36].

### 5.3. Cardiac Pacing

Atrioventricular block is a frequent presenting manifestation of CS, and permanent device implantation remains indicated even when conduction recovers following immunosuppression [25]. The rationale rests on three observations. First, recovery of atrioventricular conduction is unpredictable: FDG uptake in the region of the atrioventricular node has been suggested to predict response to corticosteroids, whereas late gadolinium enhancement without active inflammation is associated with poor response [37]. Second, recovery may be transient, with recurrence of block as inflammation relapses or as fibrosis progresses. Third, and most importantly, the presence of conduction disease identifies a population at substantial risk of ventricular arrhythmia independent of left ventricular function—high-grade block is not simply a bradycardic problem but a marker of septal granulomatous involvement adjacent to arrhythmogenic substrate.

This last consideration determines the choice of device. Because patients requiring pacing for CS-related block carry a substantial risk of sudden cardiac death even with preserved left ventricular function and no documented ventricular arrhythmia, current consensus favours implantation of a defibrillator with pacing capability rather than a pacemaker alone [17,25,38]. A subcutaneous defibrillator is generally unsuitable in this population, given the frequency of conduction disease and the consequent need for bradycardia support.

When programming detection and therapy zones, it should be borne in mind that atrioventricular block may resolve under corticosteroid therapy, restoring rapid conduction of atrial arrhythmias and creating a risk of inappropriate therapy [25].

**The site of ventricular pacing.** Beyond the choice between a pacemaker and a defibrillator, the pacing site itself warrants consideration in this population. In a prospective single-centre analysis of 40 CS patients presenting with advanced conduction disease and an initial LVEF above 40%, 26 (65%) sustained a right ventricular pacing burden exceeding 40%, and half of these (13 of 26) developed pacing-induced cardiomyopathy over a median of 23 months. This 50% incidence contrasts sharply with the 5–13% reported in unselected populations with comparable pacing burden [39].

The only variable distinguishing those who developed pacing-induced cardiomyopathy was the extent of septal scar on FDG-PET (summed rest score 3.2 ± 3.3 versus 0.5 ± 0.7; *p* = 0.011), with a score of 4 or more showing 100% specificity for its development; total and lateral scar burden did not differ. This finding is mechanistically coherent, since pacing-induced cardiomyopathy arises from ventricular dyssynchrony and a septum already compromised by granulomatous scarring is less able to accommodate the additional dyssynchrony that right ventricular pacing imposes. Of the 13 affected patients, seven were upgraded to biventricular pacing and all met the criteria for response—a higher rate than the approximately 50% reported in pooled CS series [39].

These observations argue for anticipating the consequences of chronic right ventricular pacing at the time of initial implantation rather than awaiting functional decline, particularly in patients with demonstrable septal scar, in whom resynchronization may be justified from the outset irrespective of baseline ejection fraction.

Conduction system pacing offers a further alternative. In the first reported series of left bundle branch area pacing in CS, comprising 19 patients of whom over half had septal involvement on CMR or PET, implantation succeeded in every case without acute complication, and capture threshold, R-wave amplitude and impedance remained stable over a median follow-up of 437 days. Notably, pacing parameters did not differ according to the presence of septal involvement [40]. Among the four patients with left bundle branch block, QRS above 130 ms and LVEF below 50%, ejection fraction improved from 27 ± 17% to 50 ± 12%, although the small numbers preclude firm conclusions.

These data are reassuring as to feasibility, but the cohorts are small, and the question of how conduction system pacing leads behave in patients implanted during active septal inflammation, before immunosuppression has taken effect, remains open [39]. The choice of pacing strategy should therefore be individualised according to anticipated pacing burden, ventricular function, and the distribution and activity of myocardial involvement. The indications for permanent pacing in this setting are summarised in Table 3.

### 5.4. Cardiac Resynchronization Therapy (CRT)

Heart failure complicating CS is frequently accompanied by mechanical dyssynchrony—between atrium and ventricle, between the two ventricles, or within the left ventricle itself—arising from intraventricular conduction delay. Cardiac resynchronization therapy aims to correct this, slowing the progression of heart failure and improving prognosis [41]. Ejection fraction and QRS duration on the surface electrocardiogram are the principal predictors of response; Japanese practice defines a wide QRS as 120 ms or more [42].

Evidence supporting CRT benefit specifically in CS patients remains limited, with response rates lower than in non-ischemic cardiomyopathy of other etiologies [42]. Consequently, the indications for CRT in these patients are defined on the basis of the “*Guidelines for Non-Pharmacotherapy of Cardiac Arrhythmias*” proposed by the Japanese Circulation Society. Resynchronization (CRT) is recommended for patients in NYHA ambulatory class III or class IV despite optimal medical treatment, with a left ventricular ejection fraction (LVEF) ≤ 35%, a QRS interval ≥ 120 ms and sinus rhythm, and should be considered in case of atrial fibrillation or if a pacemaker is indicated with frequent ventricular pacing [27].

A Mayo Clinic series spanning 2000 to 2021 examined 55 patients receiving resynchronization therapy, in whom CS was proven in 18, probable in 21 and presumed in 16. Two-thirds of implants (67.3%) were device upgrades rather than de novo procedures, so intrinsic QRS morphology could be characterised in only a minority. No significant improvement was observed overall, either in left ventricular function (mean ejection fraction 34.8% at baseline versus 37.7% at six months) or in end-diastolic dimension (58.5 mm versus 57.5 mm). The authors attributed this limited response to the extent of myocardial scarring and fibrosis present in advanced disease [43].

### 5.5. Catheter Ablation of Ventricular Arrhythmias in Cardiac Sarcoidosis

**Indications.** Catheter ablation is indicated in patients with ventricular tachycardia refractory to corticosteroids and antiarrhythmic drugs, in those intolerant of pharmacological therapy, and in patients presenting with electrical storm [27].

**The arrhythmogenic substrate.** Ventricular tachycardia in CS arises from a patchy, heterogeneously distributed substrate that differs from the confluent subendocardial scar of ischaemic cardiomyopathy. Granulomatous involvement preferentially affects the basal septum and the mid-myocardial and subepicardial layers of the left ventricle, with frequent right ventricular participation. As a consequence, multiple ventricular tachycardia morphologies are typically inducible in the same patient, reflecting several distinct reentrant circuits rather than a single dominant one, and complete elimination of all inducible ventricular tachycardia is often unachievable.

This anatomical distribution has direct procedural implications. Endocardial mapping of both ventricles is frequently required, and epicardial mapping and ablation are necessary in a substantial proportion of cases [44]. Bipolar voltage mapping samples predominantly the endocardial layer and may therefore underestimate substrate extent when involvement is mid-myocardial or epicardial. Unipolar voltage mapping, which has a greater depth of field, has been proposed as a complementary approach in this setting, although data specific to CS remain limited. Similarly, integration of CMR- or PET-derived scar and inflammation maps with the electroanatomical map may assist substrate definition where intramural involvement is suspected, but this approach has not been systematically validated in CS cohorts.

**Timing relative to inflammatory activity.** Active myocardial inflammation influences ablation outcome through several mechanisms. Inflammatory cytokines promote triggered activity and abnormal automaticity, generating arrhythmias that are not substrate-dependent and therefore not amenable to substrate modification [44,45]. Myocardial oedema limits the depth of thermal lesion formation, reducing the likelihood of transmural injury at the intended target. The substrate itself remains dynamic, so that a circuit successfully eliminated during active inflammation may re-emerge, or new circuits may develop, as the inflammatory process evolves.

The presence of active inflammation on FDG-PET at the time of ablation is associated with an increased risk of ventricular tachycardia recurrence. Accordingly, treatment of the active inflammatory phase with immunosuppression before attempting catheter ablation is recommended wherever the clinical situation permits [44,45]. In patients with incessant ventricular tachycardia or electrical storm this sequencing may not be feasible, and ablation is undertaken concurrently with immunosuppression, accepting a higher expected recurrence rate.

**Procedural endpoints and outcomes.** Acute procedural success—most commonly defined as non-inducibility of clinical ventricular tachycardia—is achieved less consistently than in ischaemic cardiomyopathy, and complete non-inducibility of all morphologies is an unrealistic endpoint in many patients. Elimination of the clinical ventricular tachycardia, with acceptance of persisting inducibility of non-clinical morphologies, is a more attainable objective.

In the largest multicentre analysis available, comprising 158 patients with CS-associated ventricular tachycardia undergoing catheter ablation, antiarrhythmic drug requirement and defibrillator shocks were significantly reduced, and ventricular tachycardia storm was eliminated in 82% of patients presenting with that syndrome. Left ventricular dysfunction and active myocardial inflammation were both associated with adverse long-term outcome after ablation [45]. Among patients with LVEF below 50%, survival free of ventricular tachycardia recurrence, transplantation or death was 48% at one year and 36% at two years; corresponding figures in patients with active inflammation were 52% and 43% [45].

A more recent propensity-matched analysis compared periprocedural and longer-term outcomes between patients with and without CS undergoing ventricular tachycardia ablation. Immediate periprocedural complication rates were comparable between groups. However, CS was associated with higher incidences of pericarditis, of heart failure decompensation at one and five years, and of defibrillator shocks at five years [46]. These findings support ventricular tachycardia ablation as a reasonable therapeutic option in CS while indicating that these patients warrant closer post-procedural surveillance than their non-CS counterparts.

**Repeat procedures and adjunctive therapy.** Given the evolving nature of the substrate, ventricular tachycardia recurrence after an initially successful procedure is common, and repeat ablation is required in a substantial minority of patients. Antiarrhythmic drug therapy is generally continued after ablation rather than withdrawn [36]. Persistent recurrence despite optimised immunosuppression, antiarrhythmic therapy and repeat ablation should prompt consideration of advanced heart failure therapies, including evaluation for transplantation.

### 5.6. The Wearable Cardioverter-Defibrillator

The wearable cardioverter-defibrillator (WCD) may have a role during the acute inflammatory phase, when the risk of sudden death is elevated but the need for permanent device therapy has not yet been determined. Three scenarios are relevant: while awaiting the response of ventricular function to immunosuppression; during the assessment interval before a definitive ICD decision; and as a bridge in patients temporarily unsuitable for implantation, such as those with active infection.

Evidence specific to CS is limited. A retrospective analysis of 46 CS patients prescribed a WCD, with a median left ventricular ejection fraction of 30%, reported eleven ventricular tachyarrhythmia episodes in ten patients (22%), with 100% first-shock conversion success and 100% survival at 24 h after treated events; median daily wear time was 23.6 h [47]. These findings derive from a manufacturer database and a small cohort, and should be interpreted accordingly; no randomised data exist in this population.

The WCD should therefore be regarded as a temporising strategy in selected patients rather than an established component of care, with the decision individualised according to arrhythmic risk, expected time to definitive assessment, and patient adherence.

Beyond the acute therapeutic decisions discussed above, the progressive and fluctuating nature of CS requires ongoing rhythm surveillance to detect events that may modify subsequent management.

## 6. Risk Stratification for Sudden Cardiac Death and ICD Indications

After pulmonary disease, cardiac involvement accounts for the greatest share of sarcoidosis-related mortality, and most of those deaths are sudden and arrhythmic in origin.

Defibrillator implantation remains the most effective intervention available for preventing sudden death in CS. Selection of candidates rests on a series of expert consensus documents, whose purpose is to separate patients whose arrhythmic risk justifies a device from those in whom it does not.

Both the 2022 ESC guidelines and the 2017 AHA/ACC/HRS document assign a Class I indication to two groups [29,48]:

Patients with sustained ventricular tachycardia or survivors of cardiac arrest.

Patients with a left ventricular ejection fraction (LVEF) ≤ 35%.

The ESC document extends a Class IIa indication to several further situations: patients with an ejection fraction above 35% who already require a permanent pacemaker; patients with an ejection fraction between 35% and 50% together with limited late gadolinium enhancement in whom sustained monomorphic ventricular arrhythmia is inducible at electrophysiological study; and patients with an ejection fraction above 35% in whom substantial myocardial enhancement persists once acute inflammation has resolved [29].

The 2017 AHA/ACC/HRS document considers implantation reasonable (Class IIa) in patients with an ejection fraction of 35% or above when any of the following is present [48]:

Presence of syncope.

Evidence of myocardial scarring via CMR or PET.

Positive result of an electrophysiological study (EPS).

Indication for a permanent pacemaker.

In this population a transvenous system offering bradycardia support is preferred over a subcutaneous device, given how frequently conduction disease coexists.

A practical caveat applies to device programming: atrioventricular block may recover under corticosteroid therapy, restoring rapid atrioventricular conduction of atrial arrhythmias and exposing the patient to inappropriate shocks [25].

CMR contributes substantially to prognostic assessment and to the decision to implant. Coleman and colleagues pooled 706 patients with established or suspected CS who had undergone CMR. The presence of late gadolinium enhancement was associated with markedly higher odds of arrhythmic events—ventricular arrhythmia, sudden death or appropriate device therapy—and of all-cause mortality (odds ratio 10.74, *p* < 0.00001). The association was stronger still in the subgroup with an ejection fraction of 50% or more (odds ratio 19.43, *p* < 0.00001). Annualised event rates were 11.9% among patients with enhancement compared with 1.1% among those without [49].

Programmed ventricular stimulation offers a means of refining risk assessment in patients who fall short of conventional device criteria. Its use in patients with CS, an ejection fraction above 35% and neither arrhythmic symptoms nor documented arrhythmia carries a Class IIa recommendation in both the 2017 AHA/ACC/HRS and 2016 JCS documents [27,48].

The adequacy of these consensus-based criteria has recently been challenged directly. In a multicentre study of 1514 patients with histologically supported sarcoidosis who underwent CMR for suspected cardiac involvement and were not candidates for secondary prevention, 84 experienced fatal or life-threatening ventricular arrhythmia over a median follow-up of 4.5 years. A high-risk CMR phenotype identified patients with 5- and 10-year event incidences of 24.0% and 35.0%, whereas low-risk phenotypes carried incidences of 0.7% and 2.6%. CMR phenotyping outperformed societal recommendations in discriminative accuracy, with areas under the curve of 0.861 and 0.776 for 5- and 10-year outcomes respectively [50].

These findings carry a direct implication for practice: an imaging-based phenotype may identify candidates for primary prevention more accurately than the criteria currently in use, both by capturing high-risk patients who fall outside those criteria and by sparing devices in patients whose predicted event rate is very low. The study is retrospective and observational, and prospective validation is required before guidelines are revised; nevertheless, it represents the strongest evidence to date that current ICD criteria in CS are imperfect, and it reinforces the argument for individualised assessment rather than mechanical application of thresholds.

ILLUMINATE-CS is a Japanese multicentre retrospective registry of 512 patients, distinguished from earlier cohorts by restricting inclusion to cases meeting either the 2014 HRS or 2016 JCS criteria. Over a median follow-up of three years, adverse event rates were high and driven principally by fatal ventricular arrhythmia. Four independent predictors emerged on multivariate analysis: previous ventricular tachycardia or fibrillation, elevated B-type natriuretic peptide (BNP), reduced ejection fraction, and prior radiofrequency ablation for ventricular tachycardia [10].

Two retrospective cohorts have proposed quantitative LGE thresholds for risk stratification. Nabeta et al. reported that involvement of four or more left ventricular segments, according to the 17-segment model, was associated with a significantly increased risk of adverse events [10]. Kazmirczak et al., studying 290 patients with biopsy-proven sarcoidosis followed for a composite of significant ventricular arrhythmia or SCD, found that an LGE burden exceeding 5.7% improved specificity for identifying patients at high risk of sudden death among those with ejection fraction above 35% [51].

These thresholds should not be regarded as validated or interchangeable. They derive from different cohorts with differing inclusion criteria, endpoints and quantification methods, and neither has been prospectively validated. Segment counting and percentage burden are distinct metrics that are not directly convertible, and LGE quantification itself is sensitive to the thresholding algorithm employed. At present, LGE extent is best used as a continuous marker of risk contributing to individualised assessment, rather than as a dichotomous decision rule.

## 7. Recent Advances and Future Perspectives

Recent advances include hybrid PET-MRI imaging and specific biomarkers for precise monitoring. Therapeutic innovations, such as JAK/STAT inhibitors and monoclonal antibodies, show promising potential. Registries such as ILLUMINATE-CS have begun collecting data from large cohorts of patients to better understand the natural history of cardiac sarcoidosis (CS), identify relevant prognostic biomarkers, and evaluate the efficacy of different therapeutic strategies.

Importantly, the first randomised controlled trial in cardiac sarcoidosis is now underway. CHASM CS-RCT compares low-dose prednisone combined with methotrexate against standard-dose prednisone as initial therapy in patients with active clinically manifest disease—defined by advanced conduction system disease, significant sinus node dysfunction, ventricular arrhythmia, or ventricular dysfunction. The trial tests the hypothesis that the combination will prove non-inferior in efficacy while reducing corticosteroid-related toxicity and improving quality of life [52]. Recruitment is ongoing with completion anticipated in late 2026. Its results will constitute the first randomised evidence in this field and may substantially revise current first-line treatment recommendations, which rest entirely on observational data and expert consensus.

In parallel, emerging research is exploring the application of artificial intelligence (AI) in analysing ECGs and other clinical data to enhance early detection of cardiac involvement in sarcoidosis and improve risk stratification, with a recent multimodal model developed specifically to predict sudden cardiac death in sarcoidosis, in a field where current guidance relies predominantly on left ventricular ejection fraction [53].

Despite these advances, sudden cardiac death (SCD) risk stratification in CS remains a major clinical challenge. Several unanswered questions persist:

How should asymptomatic patients with isolated cardiac sarcoidosis and preserved left ventricular ejection fraction be managed?

How can we prevent SCD while avoiding unnecessary use of implantable cardiac devices?

These unresolved issues highlight the need for prospective studies and updated clinical algorithms to guide evidence-based decision-making.

## 8. Phase-Dependent Arrhythmic Profile

The arrhythmic profile of CS differs according to whether the disease is in its inflammatory or its fibrotic phase, and this distinction carries direct consequences for treatment: arrhythmias arising during active inflammation may respond to immunosuppression, whereas those arising from established scar generally require device therapy or ablation. The principal differences between the two phases are summarised in Table 4.

## 9. Conclusions

CS remains a demanding diagnosis with the potential for a fatal outcome, presenting through a broad range of arrhythmic manifestations that call for individualised, multidisciplinary care. Multimodal imaging—CMR with late gadolinium enhancement together with FDG-PET—combined with continuous rhythm monitoring and electrophysiological assessment has appreciably sharpened early risk stratification for sudden death. Recognising high-risk patients permits timely defibrillator implantation, optimisation of immunosuppression, and catheter ablation where indicated. Important gaps nonetheless remain: no prospectively validated risk algorithm exists; management is uncertain in patients with preserved ejection fraction and intermediate scar burden; and the arrhythmic substrate evolves unpredictably under treatment. Future work should prioritise completion of the randomised trial programme now underway and the establishment of prospective multicentre registries able to define evidence-based thresholds for sudden death risk, evaluate novel biomarkers including AI-assisted electrocardiographic analysis and hybrid PET-CMR, and validate emerging immunomodulatory strategies. Close collaboration between electrophysiologists, imaging specialists, pulmonologists and rheumatologists remains essential to improving outcomes in this population.

## Figures and Tables

**Table 1:** ECG and ambulatory monitoring findings suggestive of cardiac sarcoidosis [13].

ECG Abnormalities	Ambulatory Monitoring Abnormalities
AV block Bundle branch block Supraventricular arrhythmia Pathological Q waves Fragmented QRS	Premature ventricular complexes (>10/h) Ventricular tachycardia, sustained or non-sustained Frequent premature atrial complexes or atrial tachycardia

Note. AV, atrioventricular; QRS, QRS complex. Adapted from [13].

**Table 2:** Antiarrhythmic drug recommendations for tachyarrhythmias in cardiac sarcoidosis, adapted from the JCS 2016 guidelines [27].

**Class**	**Indications**	**Level of Evidence**
Class I	No agent currently meets criteria for a Class I recommendation.	
Class IIa	Beta-blockers. Amiodarone, sotalol, or Vaughan-Williams class Ib agents when ventricular tachycardia is refractory to control.	6, Grade C1
Class IIb	Amiodarone, sotalol, or verapamil once ventricular tachycardia is adequately controlled. Class Ia or Ic agents when ventricular tachycardia remains refractory despite alternatives.	6, Grade C2
Class III	Class Ia or Ic agents in adequately controlled ventricular tachycardia.	6, Grade D

Recommendation classes follow the JCS system: Class I, treatment considered useful and effective; Class IIa, evidence or opinion favouring usefulness; Class IIb, usefulness less well established; Class III, treatment not advised and potentially harmful. Evidence grades: C1, consensus derived from expert opinion and small observational series; C2, consensus based on lower-quality or extrapolated data; D, evidence indicating potential harm, with treatment discouraged. Antiarrhythmic drug classes refer to the Vaughan-Williams classification. No agent currently carries a Class I recommendation in cardiac sarcoidosis, reflecting the absence of randomised evidence in this population.

**Table 3:** Indications for permanent pacing in atrioventricular block complicating cardiac sarcoidosis, adapted from the JCS 2016 guidelines [27].

Class	Indications	Level of Evidence
Class I	Second-degree, high-grade or complete AVB accompanied by symptomatic bradycardia. High-grade or complete AVB with marked bradycardia, or with prolonged ventricular pauses on waking.	6, Grade B
Class IIa	Persistent complete AVB in the absence of symptoms. Asymptomatic second-degree or high-grade AVB in the presence of any of the following: block situated at or distal to the His bundle; progressive cardiomyopathy attributable to the bradycardia; or atrioventricular conduction that fails to improve, or deteriorates, with exercise or atropine. Bradycardia-related symptoms in first-degree AVB where the block lies at or below the His bundle.	6, Grade B/C1
Class IIb	First-degree AVB with heart failure in whom optimising the atrioventricular delay is expected to improve haemodynamics.	6, Grade C2

Recommendation classes and evidence grades are defined in the footnote to Table 2.

**Table 4:** Comparison of Arrhythmias in Acute vs. Chronic Cardiac Sarcoidosis.

Criteria	Acute Phase (Inflammatory)	Chronic Phase (Fibrotic)
**Electrophysiological Substrate**	Active myocardial inflammation, edema	Myocardial fibrosis, scar formation
**Main Arrhythmias**	High-degree AV block, NSVT, possible AF	Sustained VT, VF, irreversible AV block
**Arrhythmia Evolution**	Often reversible with anti-inflammatory treatment	Often irreversible, requiring pacemaker/ICD
**Primary Treatment**	Corticosteroids, immunosuppressants	ICD, pacemaker, antiarrhythmics, ablation
**Monitoring**	Holter monitoring, ILR, CMR/PET follow-up	ILR, Holter monitoring, imaging to detect late arrhythmias

Note. The distinction between inflammatory and fibrotic phases is conceptual rather than absolute. In clinical practice the two processes frequently coexist within the same patient and even within adjacent myocardial segments, with active inflammation developing at the periphery of established scar. Patients may also cycle between phases, with recurrent inflammatory episodes superimposed on progressive fibrosis. This overlap has direct therapeutic implications, since a single patient may simultaneously require immunosuppression for active inflammation and device therapy or ablation for scar-related arrhythmia. The table should therefore be read as describing two poles of a continuum rather than mutually exclusive categories.

## Data Availability

No new data were created or analysed in this study. Data sharing is not applicable to this article.

## Abbreviations

The following abbreviations are used in this manuscript:

CS	Cardiac sarcoidosis
SCD	sudden cardiac death
TNF-α	tumor necrosis factor-α
TGF-β	transforming growth factor-β
IL-2	interleukin-2
IL-12	interleukin-12
AV	atrioventricular
AVB	atrioventricular block
AF	atrial fibrillation
ARVC	arrhythmogenic right ventricular cardiomyopathy
VT	ventricular tachycardia
VF	ventricular fibrillation
RBBB	right bundle branch block
LVEF	left ventricular ejection fraction
GLS	global longitudinal strain
FDG-PET	18F-fluorodeoxyglucose positron emission tomography
LGE	late gadolinium enhancement
ILR	implantable loop recorder
LV	left ventricle
RV	right ventricle
EGM	electrogram
HRS	Heart Rhythm Society
JCS	Japanese Circulation Society
AAD	antiarrhythmic drug
CRT	cardiac resynchronization therapy
ESC	European Society of Cardiology
EPS	electrophysiological study
CMR	cardiac magnetic resonance
ICD	implantable cardioverter-defibrillator
NSVT	non-sustained ventricular tachycardia
PVC	premature ventricular complex
SPECT	single-photon emission computed tomography
WCD	wearable cardioverter-defibrillator

## References

[1] Iannuzzi M.C., Rybicki B.A., Teirstein A.S. (2007). Sarcoidosis. N. Engl. J. Med..

[2] Yazaki Y., Isobe M., Hiroe M., Morimoto S., Hiramitsu S., Nakano T., Izumi T., Sekiguchi M. (2001). Prognostic determinants of long-term survival in Japanese patients with cardiac sarcoidosis treated with prednisone. Am. J. Cardiol..

[3] Gerke A.K., Hunninghake G. (2008). The immunology of sarcoidosis. Clin. Chest Med..

[4] Pour-Ghaz I., Kayali S., Abutineh I., Patel J., Roman S., Nayyar M., Yedlapati N. (2021). Cardiac Sarcoidosis: Pathophysiology, Diagnosis, and Management. Hearts.

[5] Cheng R.K., Kittleson M.M., Beavers C.J., Birnie D.H., Blankstein R., Bravo P.E., Gilotra N.A., Judson M.A., Patton K.K., Rose-Bovino L. (2024). Diagnosis and Management of Cardiac Sarcoidosis: A Scientific Statement from the American Heart Association. Circulation.

[6] Niemelä M., Uusitalo V., Pöyhönen P., Schildt J., Lehtonen J., Kupari M. (2022). Incidence and Predictors of Atrial Fibrillation in Cardiac Sarcoidosis. JACC Cardiovasc. Imaging.

[7] Pierucci N., Mariani M.V., Iannetti G., Maffei L., Coluccio A., Laviola D., Palombi M., Trivigno S., Spadafora L., Chourda E. (2026). Atrial cardiomyopathy: New pathophysiological and clinical aspects. Minerva Cardiol. Angiol..

[8] Sharma A., Okada D.R., Yacoub H., Chrispin J., Bokhari S. (2020). Diagnosis of cardiac sarcoidosis: An era of paradigm shift. Ann. Nucl. Med..

[9] Ipek E., Demirelli S., Ermis E., Inci S. (2015). Sarcoidosis and the heart: A review of the literature. Intractable Rare Dis. Res..

[10] Nabeta T., Kitai T., Naruse Y., Taniguchi T., Yoshioka K., Tanaka H., Okumura T., Sato S., Baba Y., Kida K. (2022). Risk stratification of patients with cardiac sarcoidosis: The ILLUMINATE-CS registry. Eur. Heart J..

[11] Cherrett C., Lee W., Bart N., Subbiah R. (2023). Management of the arrhythmic manifestations of cardiac sarcoidosis. Front. Cardiovasc. Med..

[12] Hoogendoorn J.C., Venlet J., Out Y.N.J., Man S., Kumar S., Sramko M., Dechering D.G., Nakajima I., Siontis K.C., Watanabe M. (2021). The precordial R′ wave: A novel discriminator between cardiac sarcoidosis and arrhythmogenic right ventricular cardiomyopathy in patients presenting with ventricular tachycardia. Heart Rhythm..

[13] Rosenfeld L.E., Chung M.K., Harding C.V., Spagnolo P., Grunewald J., Appelbaum J., Sauer W.H., Culver D.A., Joglar J.A., Lin B.A. (2021). Arrhythmias in Cardiac Sarcoidosis Bench to Bedside: A Case-Based Review. Circ. Arrhythm. Electrophysiol..

[14] Arunachalam Karikalan S., Yusuf A., El Masry H. (2024). Arrhythmias in Cardiac Sarcoidosis: Management and Prognostic Implications. J. Clin. Med..

[15] Aitken M., Chan M.V., Urzua Fresno C., Farrell A., Islam N., McInnes M.D.F., Iwanochko M., Balter M., Moayedi Y., Thavendiranathan P. (2022). Diagnostic Accuracy of Cardiac MRI versus FDG PET for Cardiac Sarcoidosis: A Systematic Review and Meta-Analysis. Radiology.

[16] Kim R.J., Chen E.L., Lima J.A., Judd R.M. (1996). Myocardial Gd-DTPA kinetics determine MRI contrast enhancement and reflect the extent and severity of myocardial injury after acute reperfused infarction. Circulation.

[17] Sharma R., Kouranos V., Cooper L.T., Metra M., Ristic A., Heidecker B., Baksi J., Wicks E., Merino J.L., Klingel K. (2024). Management of cardiac sarcoidosis. Eur. Heart J..

[18] Atterton-Evans V., Turner J., Vivanti A., Robertson T. (2020). Variances of dietary preparation for suppression of physiological 18F-FDG myocardial uptake in the presence of cardiac sarcoidosis: A systematic review. J. Nucl. Cardiol. Off. Publ. Am. Soc. Nucl. Cardiol..

[19] Cheng V.Y., Slomka P.J., Ahlen M., Thomson L.E.J., Waxman A.D., Berman D.S. (2010). Impact of carbohydrate restriction with and without fatty acid loading on myocardial 18F-FDG uptake during PET: A randomized controlled trial. J. Nucl. Cardiol. Off. Publ. Am. Soc. Nucl. Cardiol..

[20] Divakaran S., Stewart G.C., Lakdawala N.K., Padera R.F., Zhou W., Desai A.S., Givertz M.M., Mehra M.R., Kwong R.Y., Hedgire S.S. (2019). Diagnostic Accuracy of Advanced Imaging in Cardiac Sarcoidosis. Circ. Cardiovasc. Imaging.

[21] Mälkönen H., Lehtonen J., Pöyhönen P., Uusitalo V., Mäyränpää M.I., Kupari M. (2025). Endomyocardial biopsy in the diagnosis of cardiac sarcoidosis. Eur. J. Heart Fail..

[22] Maeda D., Fujimoto Y., Nabeta T., Kitai T., Naruse Y., Taniguchi T., Tanaka H., Morimoto R., Baba Y., Yoshioka K. (2026). Positive endomyocardial biopsy in cardiac sarcoidosis: Predictors and outcomes from the Japanese ILLUMINATE-CS registry. J. Card. Fail..

[23] Ezzeddine F.M., Kapa S., Rosenbaum A., Blauwet L., Deshmukh A.J., AbouEzzeddine O.F., Maleszewski J.J., Asirvatham S.J., Bois J.P., Schirger J.A. (2021). Electrogram-guided endomyocardial biopsy yield in patients with suspected cardiac sarcoidosis and relation to outcomes. J. Cardiovasc. Electrophysiol..

[24] Bakker A., Mathijssen H., Dorland G., Balt J.C., van Dijk V.F., Veltkamp M., Akdim F., Grutters J.C., Post M.C. (2022). Long-term monitoring of arrhythmias with cardiovascular implantable electronic devices in patients with cardiac sarcoidosis. Heart Rhythm..

[25] Birnie D.H., Sauer W.H., Bogun F., Cooper J.M., Culver D.A., Duvernoy C.S., Judson M.A., Kron J., Mehta D., Nielsen J.C. (2014). HRS Expert Consensus Statement on the Diagnosis and Management of Arrhythmias Associated with Cardiac Sarcoidosis. Heart Rhythm..

[26] Ploux S., Strik M., Caillol T., Ramirez F.D., Abu-Alrub S., Marchand H., Buliard S., Haïssaguerre M., Bordachar P. (2022). Beyond the wrist: Using a smartwatch electrocardiogram to detect electrocardiographic abnormalities. Arch. Cardiovasc. Dis..

[27] Terasaki F., Azuma A., Anzai T., Ishizaka N., Ishida Y., Isobe M., Inomata T., Ishibashi-Ueda H., Eishi Y., Kitakaze M. (2019). JCS 2016 Guideline on Diagnosis and Treatment of Cardiac Sarcoidosis—Digest Version. Circ. J. Off. J. Jpn. Circ. Soc..

[28] Schulz-Menger J., Collini V., Gröschel J., Adler Y., Brucato A., Christian V., Ferreira V.M., Gandjbakhch E., Heidecker B., Kerneis M. (2025). 2025 ESC guidelines for the management of myocarditis and pericarditis. Eur. Heart J..

[29] Zeppenfeld K., Tfelt-Hansen J., de Riva M., Winkel B.G., Behr E.R., Blom N.A., Charron P., Corrado D., Dagres N., de Chillou C. (2022). 2022 ESC Guidelines for the management of patients with ventricular arrhythmias and the prevention of sudden cardiac death. Eur. Heart J..

[30] Bussinguer M., Danielian A., Sharma O.P. (2012). Cardiac sarcoidosis: Diagnosis and management. Curr. Treat. Options Cardiovasc. Med..

[31] Barnabe C., McMeekin J., Howarth A., Martin L. (2008). Successful treatment of cardiac sarcoidosis with infliximab. J. Rheumatol..

[32] Brooks L., Kivlin W., Mohananey D., Sabchyshyn V., Putman M. (2025). Comparative effectiveness of disease-modifying antirheumatic drugs for patients with cardiac sarcoidosis. Rheumatology.

[33] Alipour P., Nery P.B., Beanlands R.S., Wiefels C., Tavoosi A., Boczar K., De Bortoli A., Leung E., Birnie D. (2025). Durable remission of cardiac sarcoidosis following discontinuation of methotrexate: A prospective cohort study. Respir. Med..

[34] Fazelpour S., Sadek M.M., Nery P.B., Beanlands R.S., Tzemos N., Toma M., Birnie D.H. (2021). Corticosteroid and Immunosuppressant Therapy for Cardiac Sarcoidosis: A Systematic Review. J. Am. Heart Assoc..

[35] Furushima H., Chinushi M., Sugiura H., Kasai H., Washizuka T., Aizawa Y. (2004). Ventricular tachyarrhythmia associated with cardiac sarcoidosis: Its mechanisms and outcome. Clin. Cardiol..

[36] Tokuda M., Tedrow U.B., Kojodjojo P., Inada K., Koplan B.A., Michaud G.F., John R.M., Epstein L.M., Stevenson W.G. (2012). Catheter Ablation of Ventricular Tachycardia in Nonischemic Heart Disease. Circ. Arrhythmia Electrophysiol..

[37] Yalta K., Yetkın E. (2023). Atrioventricular block in the setting of cardiac sarcoidosis: Further implications. Heart Vessels.

[38] Yodogawa K., Ohara T., Murata H., Iwasaki Y., Seino Y., Shimizu W. (2020). Detection of arrhythmogenic substrate within QRS complex in patients with cardiac sarcoidosis using wavelet-transformed ECG. Heart Vessels.

[39] Nelson D., Alharbi F., Tavoosi A., Nery P.B., Beanlands R., Birnie D. (2026). High incidence of pacing-induced cardiomyopathy in patients with cardiac sarcoidosis. Heart Rhythm.

[40] Gurin M., Litt M., Zghaib T., Peters C., Markman T.M., Luebbert J.J., Riley M.P., Shivamurthy P., Hyman M.C., Deo R. (2026). Left bundle branch area pacing in patients with cardiac sarcoidosis. JACC Clin. Electrophysiol..

[41] Cleland J.G.F., Daubert J.C., Erdmann E., Freemantle N., Gras D., Kappenberger L., Tavazzi L., Cardiac Resynchronization-Heart Failure (CARE-HF) Study Investigators (2005). The effect of cardiac resynchronization on morbidity and mortality in heart failure. N. Engl. J. Med..

[42] JCS Joint Working Group (2013). Guidelines for Non-Pharmacotherapy of Cardiac Arrhythmias (JCS 2011). Circ. J..

[43] Shabtaie S.A., Sehrawat O., Lee J.Z., Cha Y.-M., Mulpuru S.K., Kowlgi N.G., Siontis K.C., Rosenbaum A.N., Bois J.P., AbouEzzeddine O.F. (2022). Cardiac resynchronization therapy response in cardiac sarcoidosis. J. Cardiovasc. Electrophysiol..

[44] Papageorgiou N., Providência R., Bronis K., Dechering D.G., Srinivasan N., Eckardt L., Lambiase P.D. (2018). Catheter ablation for ventricular tachycardia in patients with cardiac sarcoidosis: A systematic review. EP Eur..

[45] Siontis K.C., Santangeli P., Muser D., Marchlinski F.E., Zeppenfeld K., Hoogendoorn J.C., Narasimhan C., Sauer W.H., Zipse M.M., Kapa S. (2022). Outcomes Associated with Catheter Ablation of Ventricular Tachycardia in Patients with Cardiac Sarcoidosis. JAMA Cardiol..

[46] Al Taii H., Saxena R., Morcos R., Al-Shammari A.S., Farhat K., Al Sakini A.S., Al-Wssawi A., Gaalema D., Naraynan A., Sabayon D. (2025). Outcomes of catheter ablation in cardiac sarcoidosis patients with ventricular tachycardia: A propensity score-matched retrospective analysis. J. Interv. Card. Electrophysiol..

[47] Skowasch D., Ringquist S., Nickenig G., Andrié R. (2018). Management of sudden cardiac death in cardiac sarcoidosis using the wearable cardioverter defibrillator. PLoS ONE.

[48] Al-Khatib S.M., Stevenson W.G., Ackerman M.J., Bryant W.J., Callans D.J., Curtis A.B., Deal B.J., Dickfeld T., Field M.E., Fonarow G.C. (2018). 2017 AHA/ACC/HRS guideline for management of patients with ventricular arrhythmias and the prevention of sudden cardiac death: A Report of the American College of Cardiology/American Heart Association Task Force on Clinical Practice Guidelines and the Heart Rhythm Society. Heart Rhythm.

[49] Coleman G.C., Shaw P.W., Balfour P.C., Gonzalez J.A., Kramer C.M., Patel A.R., Salerno M. (2017). Prognostic Value of Myocardial Scarring on CMR in Patients with Cardiac Sarcoidosis. JACC Cardiovasc. Imaging.

[50] Mathijssen H., Bawaskar P.H., Rochlani Y., Georgy I., Athwal P.S.S., Guo Y., Pollmann D., Markowitz J., Von Wald L., Roukoz H. (2025). Prediction of ventricular arrhythmic outcomes in suspected cardiac sarcoidosis: A comparison of cardiovascular magnetic resonance phenotyping vs. societal recommendations for implantable cardioverter-defibrillator placement. Eur. Heart J..

[51] Kazmirczak F., Chen K.A., Adabag S., von Wald L., Roukoz H., Benditt D.G., Okasha O., Farzaneh-Far A., Markowitz J., Nijjar P.S. (2019). Assessment of the 2017 AHA/ACC/HRS guideline recommendations for implantable cardioverter-defibrillator implantation in cardiac sarcoidosis. Circ. Arrhythm. Electrophysiol..

[52] Birnie D.H., Beanlands R.S.B., Nery P.B., Aaron S.D., Culver D.A., DeKemp R.A., Gula L., Ha A., Healey J.S., Inoue Y. (2019). Cardiac sarcoidosis multi-center randomized controlled trial (CHASM CS-RCT). Am. Heart J..

[53] Lai C., Yin M., Kholmovski E., Sani M.M., Gilotra N.A., Chrispin J., Trayanova N.A. (2026). Predicting sudden cardiac death in patients with sarcoidosis using a multimodal artificial intelligence model. JACC Clin. Electrophysiol..

